# Unusual Multiple Recurrences of Soft Tissue Giant Cell Tumors in a Patient Above 60 Years

**DOI:** 10.7759/cureus.59195

**Published:** 2024-04-28

**Authors:** Wazzan ALjuhani, Raed Qeretli, Faisal Alhabradi, Fay A Alotaibi

**Affiliations:** 1 Department of Orthopedic Surgery, Ministry of the National Guard-Health Affairs, Riyadh, SAU; 2 Department of Orthopedic Surgery, King Abdullah International Medical Research Center, Riyadh, SAU; 3 Department of Orthopedic Surgery, King Saud Bin Abdulaziz University for Health Sciences, Riyadh, SAU; 4 Department of Orthopedic Surgery, Prince Sultan Military Medical City, Riyadh, SAU; 5 College of Medicine, King Saud Bin Abdulaziz University for Health Sciences College of Medicine, Riyadh, SAU

**Keywords:** soft tissue, surgical excision, recurrence, giant cell tumors, case report

## Abstract

Primary giant cell tumors of soft tissues (GCT-ST) are rare neoplasms that share histopathological and immunohistochemical characteristics with osseous giant cell tumors. While GCT-ST generally exhibits a benign progression and can affect individuals of various ages, older patients may face a higher risk of recurrence and aggressive disease progression. In this case report, we present the case of a 63-year-old woman who experienced recurrent GCT-ST nine months after the complete excision of an initially localized tumor. Despite the mainstay treatment of GCT-ST being tumor-free margin surgical excision, this case demonstrates the occurrence of recurrences. The etiology of recurrence in GCT-ST remains unclear, highlighting the need for further studies and careful patient follow-up to prevent potential complications such as lung metastasis or widespread metastasis. Thus, this report aims to raise awareness of these tumors and emphasize the importance of diligent patient follow-up to facilitate early identification and management, thereby preventing potential complications such as lung or widespread metastasis.

## Introduction

Giant cell tumours of soft tissues (GCT-ST) are rare neoplasms that can occur in both deep and superficial soft tissues [1]. These tumours share histopathological and immunohistochemical characteristics with osseous giant cell tumours [1]. While GCT-ST cases typically exhibit a benign progression and commonly affect middle-aged individuals, it is uncommon for patients over 60 years to develop the condition [1,2].

Moreover, there have been only a few reported instances of local recurrence or distant metastases in this age group [1,2]. However, no differences were found between elderly and younger patients regarding tumour location, radiographic characteristics, and clinical progression when observing the behavior of GCT [3]. GCT-ST of low malignant potential is a relatively rare and unique condition that primarily develops in the extremities' superficial and deep soft tissues. It shares similarities with giant cell tumours of the bone, characterized by the presence of multinodular aggregates [1]. Although primary GCT-ST of low malignant potential does not exhibit a preference for any age group or sex, it commonly occurs in the younger age group [1]. There have been a few reported cases of this tumour occurring in the head and neck region [4]. Despite its rare occurrence, this tumour exhibits biologically benign behaviour without any aggressive features [1]. However, due to its infiltrative growth pattern, it has the potential to invade and affect delicate anatomical structures located deep within the body [2]. Early detection of recurrent GCT-ST cases is crucial to prevent potential complications such as metastasis [5]. Metastasis significantly worsens the prognosis and limits treatment options [5].

If a recurrent GCT-ST remains undetected or untreated, there is an increased risk of tumour cells spreading to distant organs or tissues, including the lungs [5]. Lung metastasis is particularly concerning, as it can compromise respiratory function and lead to significant morbidity and mortality [5]. Early identification of recurrent GCT-ST allows for timely intervention and management [6]. This may involve additional surgical excision, radiation therapy, chemotherapy, or targeted therapy [7]. Early intervention can potentially prevent or limit the spread of tumour cells, reducing the chances of metastasis and its associated complications [5]. Furthermore, careful patient follow-up plays a crucial role in monitoring for signs or symptoms of recurrence [8].

Regular clinical examinations, imaging studies, and laboratory tests are essential in detecting any changes or indicators of disease progression [9]. Close surveillance enables healthcare providers to identify recurrent tumours at an early stage when they are more likely to be localized and potentially more responsive to treatment [10]. Early detection and diligent follow-up of patients can help prevent the consequences of tumour progression through conservative management [11]. Therefore, the purpose of sharing cases that involve the early detection of tumours is to raise awareness about GCT-ST and emphasize the significance of closely monitoring patients to detect and approach them at an early stage [12]. This is crucial to prevent the spread of the tumour to the lungs or other parts of the body [12].

## Case presentation

A 63-year-old medically free female presented to our clinic with complaints of heaviness and progressive swelling in her proximal leg, accompanied by an increasing mass. We conducted a thorough assessment and evaluation of the leg mass. Initially, we performed simple tests, including a complete blood count (CBC), calcium level, and parathyroid hormone, all of which returned within normal ranges. The patient's white blood cell count (WBC) and hemoglobin levels were also normal. Subsequently, we proceeded with the initial staging, which revealed a large, localized mass in the patient's proximal leg appeared within one month. According to the patient, several new masses appeared on the anterior right thigh, and no other lesions were detected elsewhere. Following the recommended principles by musculoskeletal radiologists, we performed a biopsy of the lesion. The histopathology of the specimen confirmed the complete excision of the lesion.

**Figure 1:**
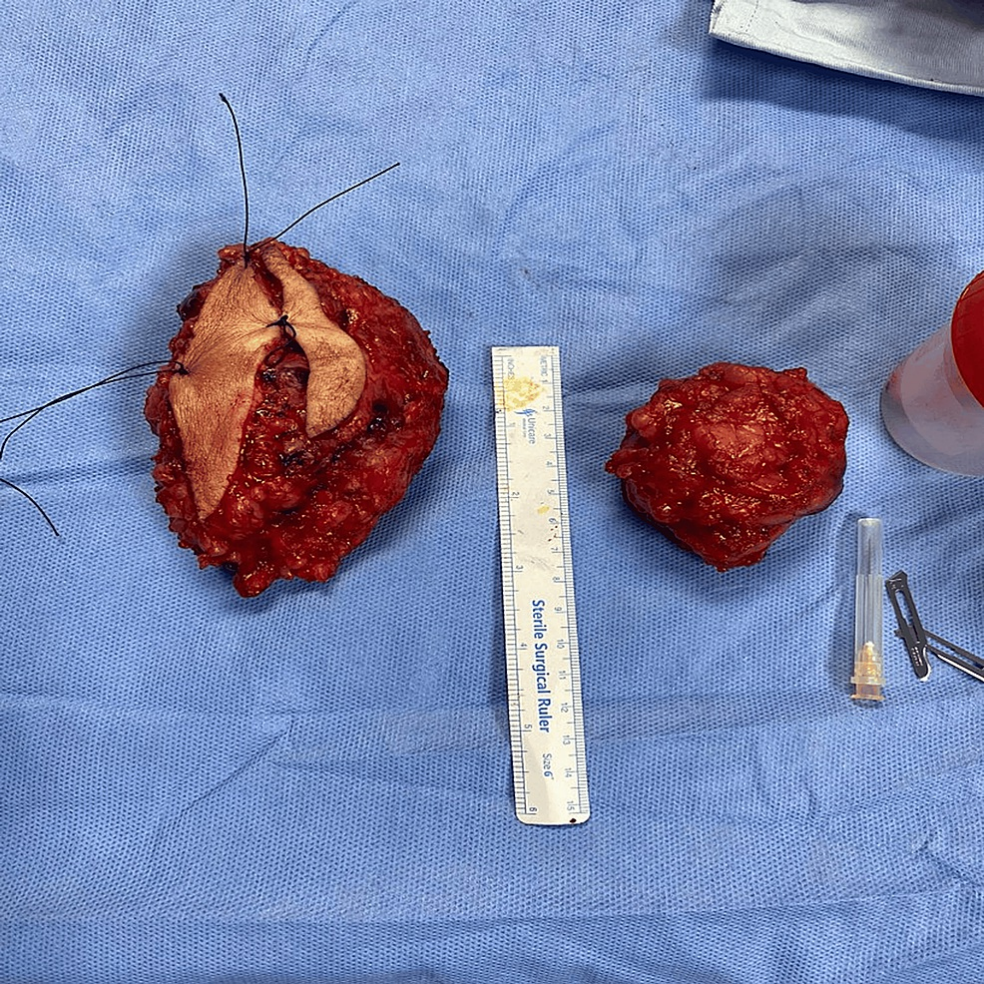
Intraoperative picture of the tumour

It exhibited a well-defined multi-nodular tumour that had infiltrated the dermis and subcutaneous fat, consisting of a combination of mononuclear cells, spindle cells, and numerous multi-nucleated osteoclast-like giant cells (OGCs). Further immunohistochemical analysis of the specimen demonstrated positive expression of P63 (in mononuclear and spindle cells) and CD163 (in mononuclear cells) while being negative for SMA, S100, and D2-40. These findings provided additional confirmation of the diagnosis of a giant cell tumor. Subsequently, the tumour was surgically removed through En Bloc resection (Figure 1).

**Figure 2:**
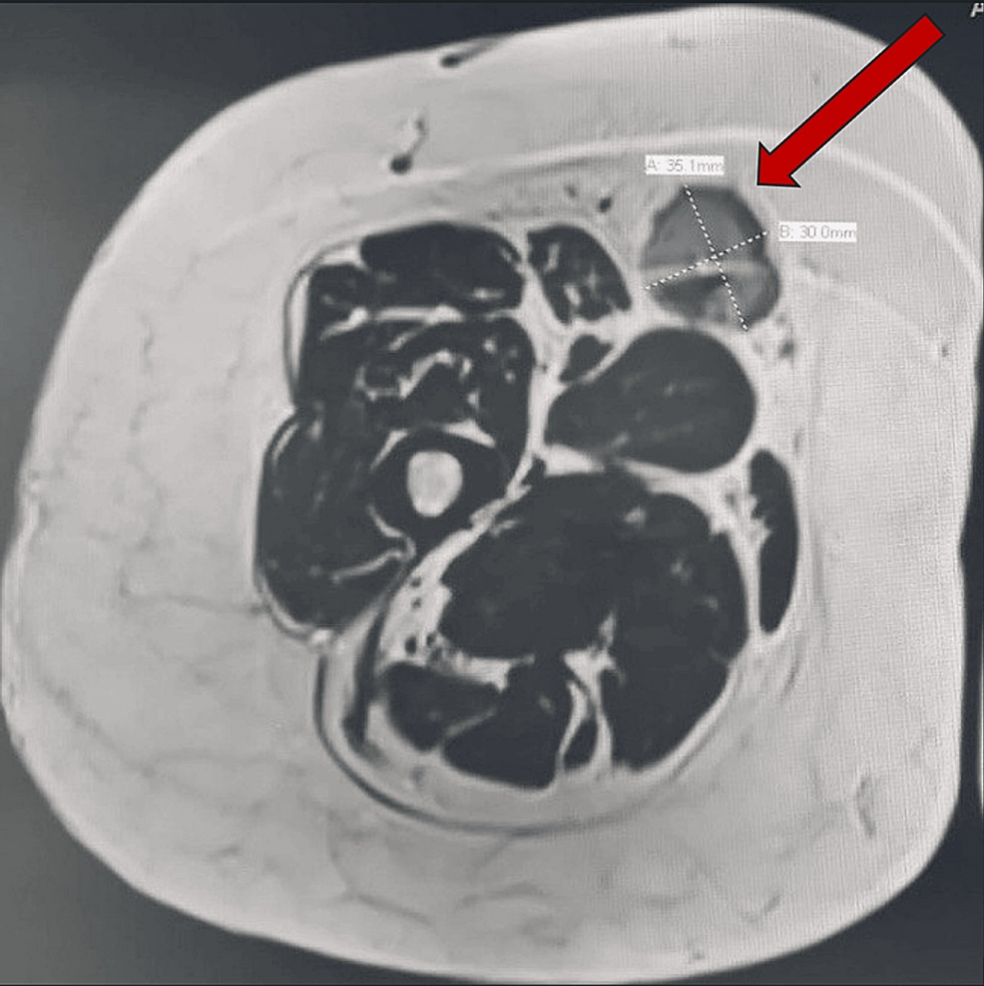
MRI showing a mass at the anterolateral right thigh

**Figure 3:**
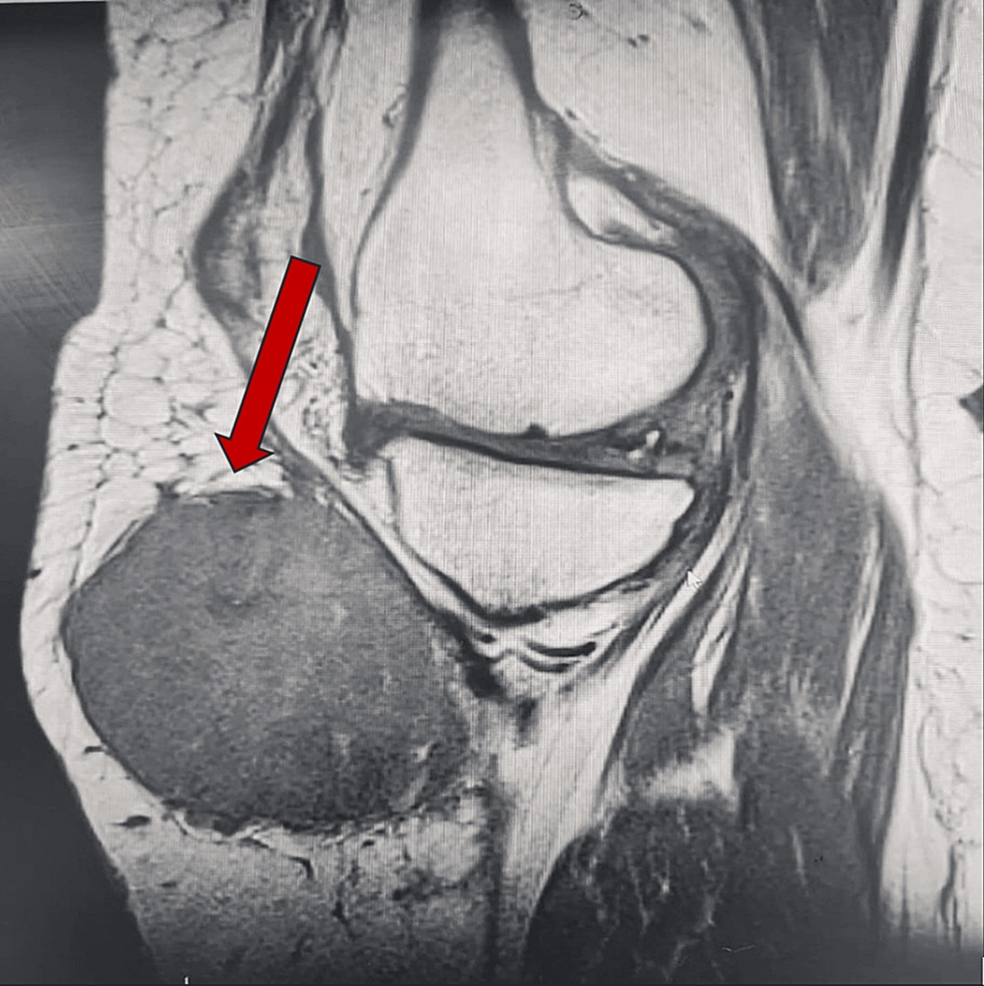
MRI Image showing a new mass on the anterior right thigh

After a period of nine months, the patient returned with complaints of pain and swelling at the site of the surgical scar. A repeat MRI of the entire lower limb revealed the presence of a new lesion adjacent to the previous tumour, as well as a small lesion in the subcutaneous area and a third lesion in the proximal thigh, away from the initial tumour (Figures 2, 3). Morphologically, the new lesion did not differ from the previous one. Consequently, the patient underwent excision of the tumour, accompanied by a gastrocnemius rotation flap and a skin graft for coverage and reconstruction.

## Discussion

GCT-ST is a rare condition, and the frequency of its occurrence in older patients is not extensively documented in existing literature. However, several studies have indicated that age can serve as a prognostic factor in other soft tissue tumors, with older patients often experiencing a more aggressive disease course and higher recurrence rates (7).

To the best of our knowledge, fewer than 20 cases of GCT-ST have been reported in patients over the age of 60 who experienced tumor recurrence. This suggests that while GCT-ST can affect individuals of various ages, the chances of recurrence or a more aggressive disease course increase when it occurs in older patients [2,5]. This may be attributed to age-related changes in the immune system, which can impact tumor surveillance and clearance [8].

GCT-ST is considered to have a benign clinical course and rare metastasis, similar to its bony counterpart. However, the rates of recurrence differ between the two. Giant cell tumors (GCTs) of bone have a higher tendency for recurrence, with rates of approximately 25% in long bones and around 50% in the axial skeleton. In comparison, GCT-ST has a much lower recurrence rate of 6.2% [2,9]. One study reported a recurrence in one out of 16 patients who underwent surgical resection of the tumor (2). Additionally, Gruccion and Enzinger reported 17 cases of recurrence, with seven of them involving lung or widespread metastasis (5).

In our case, histopathology confirmed complete excision of the initial lesion, yet there was recurrence with multiple new lesions occurring proximally. However, no lung or widespread metastasis was noted. The etiology of recurrence in GCT-ST remains unclear, but it could be attributed to metastases that were too small to detect during the preoperative evaluation and within the nine-month interval, which subsequently increased in size and presented clinically. Satellite lesions or multifocal disease can also contribute to recurrence. GCT-ST occasionally presents as multiple lesions within the same anatomical region or in different soft tissue sites. These satellite lesions may not be immediately apparent during the initial evaluation and surgical excision, leading to recurrence if they are not identified and removed (9). This highlights the need for further studies to understand the factors contributing to recurrence in GCT-ST cases and to evaluate additional management options that could prevent recurrence. Histopathological and immunohistochemical features of GCT-ST closely resemble those of bone GCT.

Both types of tumors lack atypia, pleomorphism, and atypical mitosis, but they may exhibit necrosis, metaplastic bone formation, and other features. In some cases, a bony shell may form around the tumor in both types of tumors (6). Additionally, CD68 positivity is generally observed in the osteoclast-like giant cells (OGCs) and focally in the mononuclear stromal cells in both tumor types (10). Therefore, they are believed to have a similar line of differentiation and exhibit identical clinicopathological characteristics. The mainstay treatment for GCT-ST involves complete surgical excision of the lesion with tumor-free margins to ensure a favorable prognosis with a low recurrence rate. Reports have shown lung metastasis in cases with positive surgical margins (11). Furthermore, in the context of soft tissue sarcoma, denosumab has primarily been investigated in relation to desmoid tumors. Desmoid tumors are a type of soft tissue sarcoma that arises from connective tissue cells and can occur in various parts of the body. These tumors are characterized by their locally invasive nature and propensity for recurrence. Studies have suggested that denosumab may have a role in managing aggressive or symptomatic desmoid tumors. In some cases, desmoid tumors may be associated with mutations in the *CTNNB1* gene, leading to activation of the Wnt signaling pathway.

Denosumab may interfere with this pathway and inhibit tumour growth [12]. However, in our case, the patient received six cycles of denosumab, but it showed no effect on the tumor. This might be due to the absence of histone 3 mutation in giant cells of soft tissue, which is found in giant cells of the bone. This explains why denosumab works on bone tumours but not on soft tissue tumours. Moreover, as demonstrated in our case, recurrence can still occur even with conservative surgical excision and tumor-free margins. This highlights the importance of closely monitoring patients to detect any signs of recurrence and to avoid further complications such as lung or widespread metastasis.

## Conclusions

In conclusion, early identification and diligent follow-up of patients with recurrent giant cell tumours of soft tissue (GCT-ST) are crucial in managing this condition effectively and preventing the development of metastasis and its associated complications. The timely intervention and appropriate management strategies are vital for improving treatment success rates and overall patient outcomes. Regular monitoring of patients with GCT-ST can detect recurrences or disease progression promptly, allowing for timely and individualized interventions. A multidisciplinary approach involving various specialists ensures comprehensive care tailored to the unique characteristics of each case. Treatment plans may involve a combination of surgical excision, radiation therapy, chemotherapy, or other targeted therapies, depending on the specific characteristics of the tumour. Through diligent follow-up and personalized management, we strive to optimize treatment outcomes and provide the best possible care for patients with recurrent GCT-ST.

## Figures and Tables

**Figure 4:**
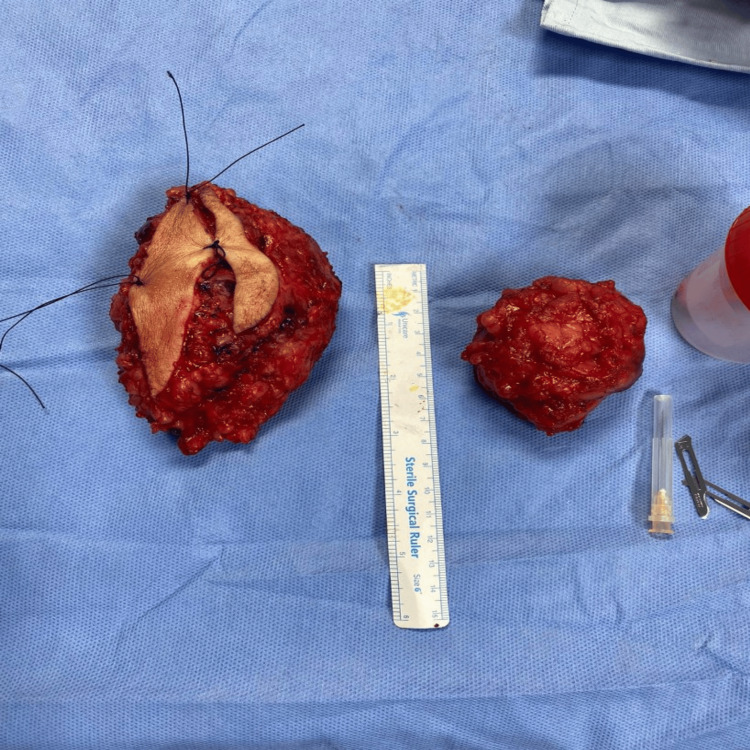
Intraoperative Picture of The Tumour

**Figure 5:**
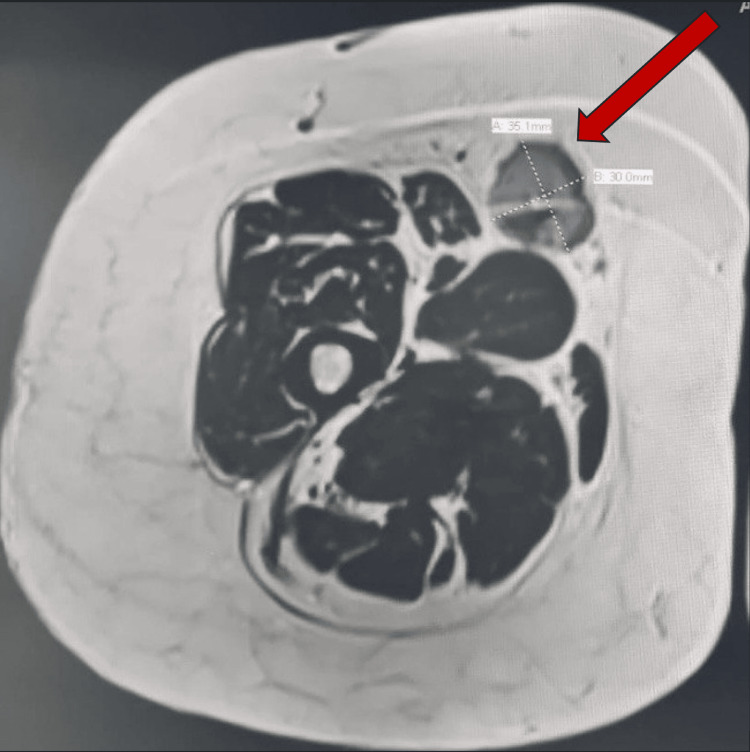
MRI showing a mass at the anterolateral right thigh

**Figure 6:**
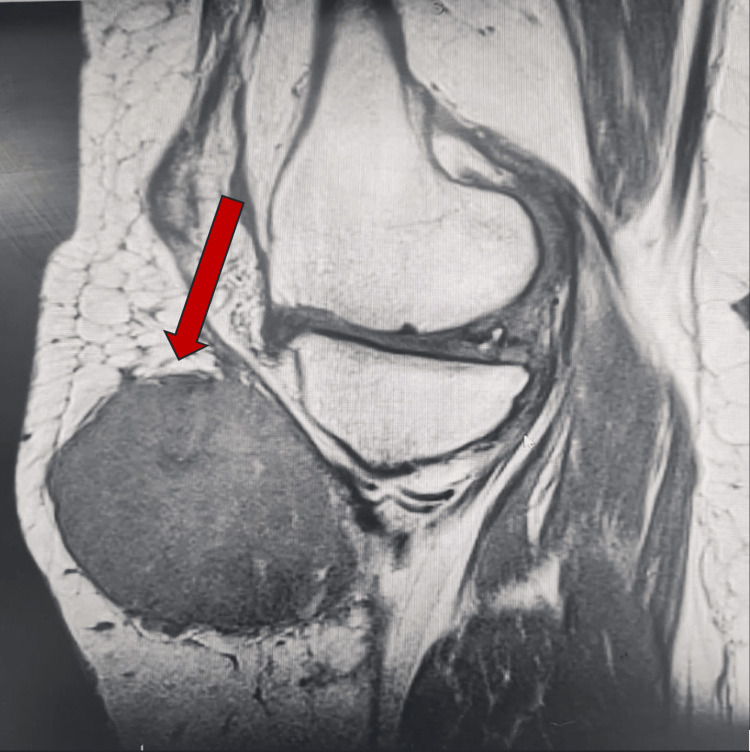
MRI Image showing new mass on the anterior right thigh
